# Impact of Preeclampsia on Long-Term Kidney Function in a Low-Resourced Setting

**DOI:** 10.1016/j.ekir.2025.11.014

**Published:** 2025-11-20

**Authors:** Bianca Davidson, Kate Bramham, Kathryn Manning, Ayesha Osman, Abigail Blumenthal, Katherine Clark, Alice Beadmore-Gray, Brian Rayner, Nicola Wearne, Erika S.W. Jones

**Affiliations:** 1 Division of Nephrology and Hypertension, Kidney and Hypertension Research Unit, University of Cape Town, Cape Town, South Africa; 2 Department of Women and Children’s Health, Faculty of Life Course and Population Sciences, King’s College London, London, UK; 3 Department of Surgery Statistics, University of Cape Town, Cape Town, South Africa; 4 Department of Obstetrics and Gynaecology, University of Cape Town, Cape Town, South Africa

**Keywords:** albuminuria, chronic kidney disease, preeclampsia

## Abstract

**Introduction:**

Hypertensive disorders of pregnancy (HDPs) affect approximately 10% of pregnancies, with disproportionate morbidity and mortality in low- and middle-income countries. Although long-term cardiovascular and kidney sequelae of preeclampsia are established in high-income settings, data from low- and middle-income countries remain scarce. We assessed incident hypertension, kidney dysfunction, and albuminuria following preeclampsia and associated risk factors.

**Methods:**

A prospective cohort study was conducted at Groote Schuur Hospital, South Africa, from January 2020 to October 2024. Clinical, demographic, and biochemical data were collected at delivery, first follow-up, and at 1- and 2-year visits. Logistic regression was used to assess predictors of hypertension, reduced estimated glomerular filtration rate (eGFR), and albuminuria, at time points stated above.

**Results:**

Of 241 women enrolled, 93 and 34 completed 1- and 2-year follow-up, respectively. The median age was 30 years (interquartile range [IQR]: 25–35), 50% had pregnancy-related acute kidney injury (AKI, Pr-AKI) and liver injury. Most (65%) delivered before 34 weeks. Incident hypertension persisted in 44% at first visit, 47% at 1 year, and 45% at 2 years. Reduced eGFR (< 90 ml/min per 1.73 m^2^) occurred in 22% at first visit and 20% at 1-year. Albuminuria persisted in > 50%. Pr-AKI predicted reduced eGFR (*P* = 0.013) and albuminuria (*P* = 0.015) at 1 year. HIV predicted reduced eGFR at first visit (*P* = 0.001), whereas elevated body mass index (BMI) predicted hypertension at 1 year (odds ratio [OR]: 3.98, *P* = 0.036).

**Conclusion:**

Cardio-renal sequelae following preeclampsia were strikingly common, underscoring the need for integrated postpartum surveillance. In this cohort, Pr-AKI was an important determinant of chronic kidney disease (CKD).

## Graphical abstract

**Figure:**
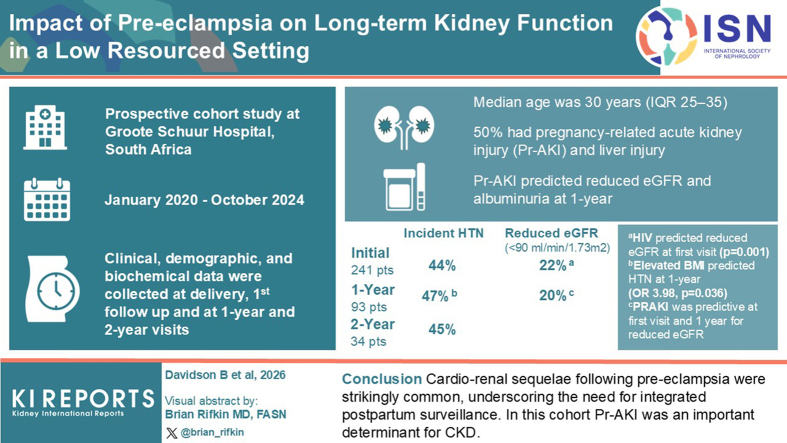

HDPs affect approximately 10% of all pregnancies; however preeclampsia incidence varies by population.^1^ The World Health Organization estimates a 7-fold higher rate of preeclampsia in low-income countries,^2^ with incidences ranging between 1.8% and 17%.^3^ Africa, the Caribbean, and Southeast Asia carry the highest HDP burden.^4^ Although studies from Africa differ in sampling and definitions used, a meta-analysis from sub-Saharan Africa reported a pooled incidence of preeclampsia of 13% (95% confidence interval [CI]: 0.12–0.14].^5^ A high mortality from HDPs is found in Africa and Latin America (17% and 22%, respectively).^6^ Contributing factors include dialysis and intensive care availability, inadequate antenatal care, and delays in detection and diagnosis.^7^

Beyond maternal mortality, there is mounting evidence of increased long-term cardiovascular and kidney risks following preeclampsia.^8^ For every death, 50 to 100 women experience significant morbidity, with maternal near-miss events now exceeding deaths.^9^^,^^10^ The most common long-term complication is early onset hypertension, with a relative risk of 3.13 (95% CI: 2.51–3.89) 2 decades post-HDP.^8^^,^^11^^,^^12^ A recent meta-analysis described a 6-fold higher risk of hypertension within 2 years of delivery,^13^ highlighting an earlier window for targeted intervention.^11^ This opportunity for intervention is essential as Africa faces a major burden of noncommunicable diseases. Hypertension prevalence in sub-Saharan Africa ranges between 30% and 50%, affecting approximately 100 million people.^13^

Robust evidence now links preeclampsia with CKD and end-stage kidney disease. Population studies have suggested a 4-fold increase in microalbuminuria at a mean of 7-years postpartum, which increases to 8-fold if preeclampsia was severe.^14^ A large prospective cohort study reported a 9-fold risk of developing CKD following preeclampsia.^15^ A meta-analysis reported a relative risk of end-stage kidney disease of 6.35 (95% CI: 2.73–14.79).^16^ AKI is also a risk factor for CKD, and for future preeclampsia.^17^^,^^18^

Despite the high burden of preeclampsia in Africa, to our knowledge, no studies have reported on persistent albuminuria and only 1 has described long-term kidney dysfunction after preeclampsia. Understanding these complications is critical in low-income settings, where access to interventions, including dialysis, is limited.^7^^,^^19^ Our study aimed to determine kidney outcomes following severe preeclampsia in a South African setting. The primary objective was to describe the prevalence of sustained hypertension, persistent albuminuria, and kidney dysfunction at 3 months, 1 and 2 years in women with and without Pr-AKI. The secondary objectives were to identify predictors of the primary outcomes, to support future risk stratification and improve clinical care possibilities.

## Methods

This prospective observational cohort study was conducted at Groote Schuur Hospital, Cape Town, South Africa using data from the postpartum hypertension registry (HREC R038/2017) from 2020 to 2024. Ethics approval was granted by the Human Research Ethics Committee at the University of Cape Town (HREC 856/2023). The inclusion criteria were women who experienced preeclampsia and attended the postpartum clinic. Because of staffing constraints, only women with complicated preeclampsia were followed-up at this clinic. Complicated preeclampsia we defined as preeclampsia with > 1 complication including PrAKI, another organ involvement, preterm delivery (< 37 weeks), or fetal loss. Exclusion criteria were those failing to meet the definition of preeclampsia, those with evidence of preexisting CKD, and those with previously known or proven glomerulonephritis during the current pregnancy or postpartum.

### Data Collection

Baseline characteristics were collected at the time of delivery. Demographic and clinical data included age, gravidity, parity, and preexisting conditions (HIV, previous hypertension, preeclampsia in a previous pregnancy, and diabetes mellitus). Complications included increased liver enzymes; hematological complications; hemolysis, elevated liver enzymes, and low platelets [HELLP]; eclampsia; placental abruption; pulmonary edema; and Pr-AKI.

Delivery details (mode, complications, postpartum hemorrhage, hysterectomy) and baby outcomes (alive, APGARS, birth weight, and centile) were recorded. Laboratory data at delivery include creatinine, potassium, uric acid, proteinuria, full blood count, peripheral blood smear, and liver profile. Birth centiles were calculated using the Fetal Medicine Foundation birth centile calculator.^20^

Routine follow-up visits occurred at 3 to 6 months, 1- and 2-year. Clinical data included office blood pressure [BP] and ambulatory BP (if indicated), electrocardiogram (first visit and annually), anthropometry (waist circumference, neck measurement, height, and weight) and clinical evidence of heart failure. Laboratory data included kidney function (urea, creatinine and eGFR,^21^ and spot urine for albumin-creatinine ratio).

### Definitions

Preeclampsia was defined as hypertension developing after 20 weeks’ gestation with ≥ 1 of the following: proteinuria, organ dysfunction (neurological, pulmonary edema, hematological complication, Pr-AKI, or liver injury) and/or uteroplacental dysfunction (placental abruption, fetal growth restriction, umbilical artery abnormalities, intrauterine fetal death).^22^ HELLP syndrome included evidence of hemolysis (fragments on peripheral blood smear, low haptoglobin, rise in bilirubin, and an unexplained drop in hemoglobin) with an accompanying drop in platelets (< 150∗10^9^/L) and a rise in liver enzymes (alanine transaminase or aspartate transaminase > 40 U/l).^22^ Superimposed preeclampsia was defined according to the International Society for the Study of Hypertension in Pregnancy guidelines.^22^

Preexisting CKD was defined by a prepregnancy eGFR < 90 ml/min per 1.73 m^2^, structural abnormalities seen on ultrasound, or kidney sizes < 9 cm with an eGFR < 90 ml/min per 1.73 m^2^. Lastly, if neither were available, creatinine on presentation intrapartum was reviewed. If this creatinine was abnormal and did not recover by the 3-month follow-up, the patient was excluded. AKI at delivery was defined by the Kidney Disease: Improving Global Outcomes criteria.^23^

### Outcome Definitions

Hypertension on follow-up was defined as an average BP ≥ 140/90 mm Hg at any visit (average of ≥ 2 stable office readings taken according to European Society of Hypertension guideline recommendations at a single visit).^24^ Incident hypertension was defined as an average BP > 140/90 mm Hg in women with no hypertension prior to their pregnancy or within the first 20 weeks intrapartum. Chronic preexisting hypertension was defined as women with hypertension prior to 20 weeks or already known with hypertension prepregnancy. Women with preexisting chronic hypertension were excluded from the analysis of outcome incident hypertension at follow-up and the regression analysis of associated factors linked to developing incident hypertension. Kidney dysfunction was defined as an eGFR < 90 ml/min per 1.73 m^2^ at any follow-up visit. Albuminuria was assessed on spot urine and stratified as normal (< 3 mg/mmol), moderately increased (3–30 mg/mmol), or severely increased (> 30 mg/mmol).^25^ Stillbirths were defined as fetal death prior to delivery, at > 24 weeks’ gestation or > 500 g birthweight. Missing data (owing to laboratory error, misplaced sample, or unperformed investigation) are clearly indicated in all tables.

### Data Analysis

All statistical analysis was performed using Stata (Version 17.1; Stata Corp, College Station, TX). Descriptive statistics were used to present the patient’s demographic and clinical characteristics. Continuous variables were presented as means (± SD) or medians (with IQR), whereas categorical variables were presented as frequencies and percentages. For comparison of categorical variables, chi-square or Fisher exact test was applied, as appropriate, whereas continuous variables were compared between groups using *t* test or Wilcoxon rank sum test. Spearman’s rank correlation was used to analyze the strength and direction between continuous variables. Risk factors for incident hypertension, kidney dysfunction (reduced eGFR < 90 ml/min per 1.73 m^2^) and albuminuria were analyzed using logistic regression analysis at first visit and first year post delivery. Clinically relevant baseline risk factors were chosen *a priori* (maternal age at delivery, HIV, chronic hypertension, prior HDP, AKI at delivery, HELLP syndrome, placental abruption, gestational age, and weight of fetus) and were included in exploratory univariable logistic analysis. Multivariable logistic analysis included variables associated with outcome, mild, moderate, or strong odds of outcome, and were adjusted for maternal age at delivery. Model goodness of fit was assessed using Hosmer-Lemeshow test.

## Results

**Figure 1:**
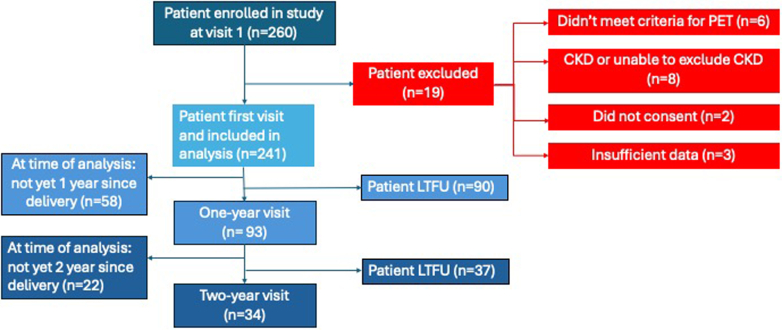
Study flow diagram. Depicts the number of patients enrolled in the registry, and the number and reasons for those excluded. CKD, chronic kidney disease; PET, preeclampsia.

Two hundred sixty women were enrolled in the study at follow-up, 241 met the inclusion criteria and were analyzed for the study (Figure 1). The median age was 30 (IQR: 25–35) years. There was a high proportion of preeclampsia with Pr-AKI in 119 of 241 (49%), liver injury in 119 of 239 (50%), low platelets in 122 of 240 (51%) and placental abruption in 28 of 239 (12%). Of the total Pr-AKI, 48 of 119 (40%) consisted of stage 2 or 3. The majority (65%) of women delivered before 34 weeks. At delivery, those with Pr-AKI had a higher systolic (*P* ≤ 0.001) and diastolic BP (*P* = 0.011), lower platelets (*P* < 0.001), and more women were likely to experience HELLP (*P* < 0.001) and placental abruption (*P* = 0.016). In Table 1, we describe the maternal and neonatal clinical and biochemical features at delivery, comparing those with and without Pr-AKI.

**Table 1:** Maternal and neonatal clinical and biochemical features at delivery, comparing those with and without AKI

Clinical variables	Total (*N* = 241)	PE without Pr-AKI (*n* = 122)	PE with Pr-AKI (*n* = 119)	*P*-value
Maternal clinical features at delivery				
Maternal age (yrs)	30 (25–35)	30 (25–30)	31 (25–36)	0.630
Prior chronic hypertension	68/241 (28%)	35/122 (29%)	33/119 (28%)	0.864
Previous HDP^a^	58/234 (25%)	30/118 (25%)	28/116 (24%)	0.820
BMI (kg/m^2^)^a^				0.350
< 25	49/227 (22%)	21/115 (18%)	28/112 (25%)	
25–30	57/227 (25%)	26/115 (23%)	31/112 (28%)	
30–35	72/227 (32%)	40/115 (35%)	32/112 (29%)	
> 35	49/227 (22%)	28/115 (24%)	21/112 (19%)	
HIV^a^	30/239 (13%)	11/120 (9%)	19/119 (16%)	0.110
DM^a^	7/238 (3%)	7/119 (6%)	0/119 (0%)	0.014
Delivery details and preeclampsia phenotyping				
Highest systolic BP (mm Hg)	167 (158–181)	164 (156–174)	174 (160–187)	< 0.001
Highest diastolic BP (mm Hg)	107 (100–112)	106 (99–110)	109 (100–114)	0.011
Peak creatinine (μmol/l)	83 (64–111)	64 (56–75)	112 (97–144)	< 0.001
Pr-AKI	119/241 (49%)			
Stage 1	71/119 (60%)		71/119 (60%)	
Stage 2	29/119 (24%)		29/119 (24%)	
Stage 3	19/119 (16%)		19/119 (16%)	
Elevated liver enzymes^a^	119/239 (50%)	44/120 (37%)	75/119 (63%)	< 0.001
Low platelets (< 150 × 10^9^/L)^a^	122/240 (51%)	44/121 (36%)	78/119 (66%)	< 0.001
HELLP^a^	96/240 (40%)	32/121 (26%)	64/119 (54%)	< 0.001
Eclampsia^a^	12/238 (5%)	3/119 (3%)	9/119 (8%)	0.140
Pulmonary edema^a^	16/239 (7%)	5/120 (4%)	11/119 (9%)	0.130
Placental abruption^a^	28/239 (12%)	8/120 (7%)	20/119 (17%)	0.016
Caesarean delivery	159/241 (66%)	79/122 (65%)	80/119 (67%)	0.690
Clinical features of fetus at delivery				
Gestational age (weeks)^a^				0.950
> 34	83/240 (35%)	42/ 121 (35%)	41/119 (34%)	
28–34	119/240 (50%)	59/121 (49%)	60/119 (50%)	
< 28	38/240 (16%)	20/121 (17%)	18/119 (15%)	
Small gestational age (birth centile)^a^				0.970
> 10 centiles	95/232 (41%)	47/116 (41%)	48/116 (41%)	
10–5 centile	30/232 (13%)	16/116 (14%)	14/116 (12%)	
< 5 centile	107/232 (46%)	53/116 (46%)	54/116 (47%)	
Stillbirths	48/234 (21%)	21/117 (18%)	27/117 (23%)	0.343

AKI, acute kidney injury; BMI, body mass index; BP, blood pressure; DM, diabetes mellitus; HDP, hypertensive disorders of pregnancy; HELLP, hemolysis, elevated liver enzymes, and low platelets; PE, preeclampsia; Pr-AKI, pregnancy-related AKI.

a Missing data reflected by denominator.

Neonatal outcomes were poor with stillbirths accounting for 48 of 234 (21%). There were 10 infants who were live-born before reaching local viability criteria, 6 of them before 24 weeks, and 8 were < 500 g at delivery. All demised in the early neonatal period. In the overall cohort, the majority were born preterm with growth restriction. The median weight was 1722 g, with a very low median birthweight centile of 7 (IQR: 1–32). A large proportion, 107 of 232 (46%), were extremely small for gestational age with a centile < 5. The median gestational age was 33 (IQR: 3036) weeks; however, 38 of 240 (16%) were severely premature and delivered before 28 weeks’ gestation.

### Outcomes of Mothers Following Preeclampsia

Because this observational study was disrupted by COVID-19, the first visit times varied. The majority (144/241, 60%) were seen between 3 to 6 months, 60 of 241 (25%) were seen just under 3 months and 37 of 241 (15%) had their first follow-up between 6 to 12 months after delivery. Annual follow-ups were scheduled as close to 1 or 2 years after delivery as possible.

**Figure 2:**
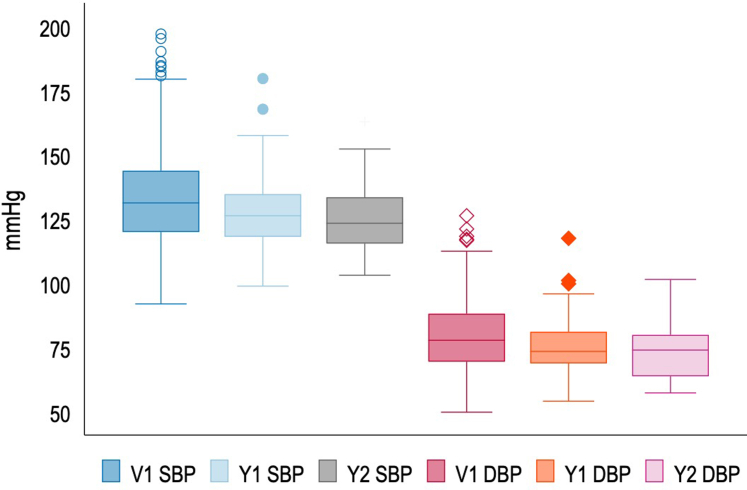
Median systolic and diastolic blood pressure and interquartile ranges**.** Depicts a box and whisper plot of median (with range) blood pressure control at first visit, 1-year and 2-year. DBP; diastolic blood pressure; SBP, systolic blood pressure; V1, visit 1; Y1, year 1 visit; Y2, year 2 visit.

Follow-up data revealed a high proportion of women with hypertension at the first visit (128/241, 53%). There were 68 women who had preexisting hypertension and 2 women did not have data. Incident hypertension at the first visit and at 1 and 2 years was 76 of 171 (44%), 28 of 60 (47%), and 9 of 20 (45%), respectively (Table 2). The highest proportion of incident hypertension was diagnosed at first visit (44%), only 4 patients were diagnosed at 1 year and none thereafter. More women with HELLP syndrome and those with preeclampsia without Pr-AKI had incident hypertension at 1 year (*P* = 0.041 and *P* = 0.046), the relevance of which is unclear (Table 2 and Supplementary Table S1). However, the proportion of women with incident hypertension was not significantly different in those with and without prior HDP, birth centile < 10 or HIV (Supplementary Table S1). In Figure 2, we demonstrate BP control on antihypertensives at the first visit and at annual follow-up. Treatment uptake was high, with 97 of 128 mothers (76%) on treatment at first visit, this increased to 51 of 55 (93%) at 1-year follow-up and 20 of 20 (100%) at 2-year follow-up (Supplementary Table S2).

**Table 2:** Description of incident hypertension, persistent albuminuria and kidney dysfunction at first visit, 1 and 2 years, comparing those with and without Pr-AKI

Follow-up data	Total cohort	PE without Pr-AKI	PE with Pr-AKI	*P*-value
First visit	241	122	119	
Incident HPT^a^	76/ 171 (44%)	40/86 (47%)	36/85 (42%)	0.580
eGFR < 90^b^	53/236 (22%)	16/120 (13%)	37/116 (32%)	< 0.001
Albuminuria^b^	132/232 (57%)	59/118 (50%)	73/114 (64%)	0.031
1st Annual visit 1	93	51	43	
Incident HPT	28/60 (47%)	19/32 (59%)	9/28 (33%)	0.046
eGFR < 90^b^	18/88 (20%)	5/49 (10%)	13/39 (33%)	0.008
Albuminuria^b^	34/74 (46%)	13/40 (32%)	21/34 (62%)	0.012
2^nd^ Annual visit 2	34	14	20	
Incident HPT	9/20 (45%)	5/9 (56%)	4/11 (36%)	0.390
eGFR < 90^b^	4/31 (13%)	1/13 (8%)	3/18 (17%)	0.460
Albuminuria^b^	18/32 (56%)	4/14 (29%)	14/18 (78%)	0.011

eGFR, estimated glomerular filtration rate (ml/min/m^2^); HPT, hypertension; PE, preeclampsia; Pr-AKI; pregnancy related acute kidney injury.

a Incident hypertension reflects new onset of HPT following preeclampsia. Analysis therefore removed those known with chronic hypertension.

b Denominators reflect the available data.

A sustained reduction in eGFR (< 90 ml/min per 1.73 m^2^) was present in 53 of 236 (22%) at first visit and 18 of 88 (20%) at 1-year (Table 2). The presence of diabetes mellitus in the cohort was low (*n* = 7) and did not confound this result, because none had a reduced eGFR at follow-up. Preexisting chronic hypertension was present in 20 of 60 (33%) with a reduced eGFR, compared with 33 of 167 (20%) in those with an eGFR > 90 ml/min per 1.73 m^2^. In those who developed Pr-AKI, there was a higher proportion of reduced eGFR and persistent albuminuria at first visit (*P* < 0.001 and *P* = 0.031, respectively) and 1-year (*P* = 0.008 and *P* = 0.012, respectively). The finding of albuminuria persisted at 2 years, despite small numbers (*P* = 0.011) (Table 2). Over half (132/232, 57%) had albuminuria at first visit and 18 of 32 (56%) at 2-years (Table 2).

A higher proportion of reduced eGFR (< 90 ml/min per 1.73 m^2^) and albuminuria was found in those with a history of prior HDP (*P* = 0.050 and *P* = 0.016, respectively) (Supplementary Table S1). A higher proportion of those with HIV had reduced eGFR at first visit (*P ≤* 0.001) and at 1 year (*P* = 0.052) (Supplementary Table S1). In Supplementary Figure S1, we categorize the degree of albuminuria with eGFR ranges at first visit and 1 year, as per the Kidney Disease: Improving Global Outcomes CKD definition.^25^

On univariate analysis, incident hypertension was associated with advanced maternal age at first visit and 1 year (*P* = 0.005 and *P* = 0.002, respectively), with raised BMI only associated at 1-year follow-up (*P* = 0.009) (Supplementary Table S3). On multivariate analysis, a raised BMI demonstrated an almost 4-fold increased risk for incident hypertension (OR: 3.98, 95% CI: 1.10–14.45, *P* = 0.036), whereas maternal age (OR: 1.12, 95% CI: 1.00–1.25; *P* = 0.051) trended toward significance, at 1 year (Table 3).

**Table 3:** Multivariate analysis of primary outcomes incident HPT, sustained reduction in eGFR and persistent albuminuria

	Incident HPT first visit			Incident HPT at 1-year		
	OR	CI	*P*-value	OR	CI	*P*-value
Maternal age at delivery	1.05	0.99–1.11	0.078	1.12	1.00–1.25	0.051
BMI > 30	1.25	0.61–2.55	0.537	3.98	1.10–14.45	0.036
Prior HDP	2.72	0.83–8.85	0.098	0.80	0.05–13.31	0.627
GA (< 34 wks)	0.92	0.46–1.85	0.824	0.87	0.24–3.18	0.833

	Reduced eGFR < 90 at first visit			Reduced eGFR < 90 at 1 yr		
	OR	CI	*P*-value	OR	CI	*P*-value
Maternal age at delivery	1.11	1.05–1.18	0.001	1.07	0.97–1.19	0.190
HIV	4.33	1.73–10.84	0.002	3.11	0.51–19.04	0.219
Prior HDP	1.63	0.74–3.61	0.225	2.59	0.68–9.89	0.163
GA (< 34 weeks)	1.35	0.61–2.98	0.461	0.96	0.25–3.65	0.957
Pr-AKI	3.25	1.56–6.77	0.002	5.44	1.44–20.58	0.013

	Persistent albuminuria at 3 mos			Persistent albuminuria at 1 yr		
	OR	CI	*P*-value	OR	CI	*P*-value
Maternal age at delivery	0.99	0.95–1.05	0.891	1.04	0.93–1.16	0.520
BMI > 30	1.47	0.79–2.76	0.227	1.43	0.40–5.10	0.583
Prior HDP	1.48	0.66–3.30	0.343	0.50	0.95–2.42	0.374
GA (< 34 wks)	0.97	0.54–1.75	0.914	1.86	0.58–5.93	0.293
Pr-AKI	2.02	1.14–3.58	0.016	3.81	1.29–11.22	0.015
Chronic hypertension	1.69	0.76–3.73	0.198	1.96	0.48–9.00	0.384

BMI, body mass index; CI, confidence interval; eGFR, estimated glomerular filtration rate; GA, gestational age at delivery; HDP, hypertensive disorders of pregnancy; HELLP; hemolysis, elevated liver enzymes, low platelets; HPT, hypertension; OR, odds ratio; Pr-AKI, pregnancy-related acute kidney injury; SGA, small for gestational age.

On univariate analysis, HIV (4-fold, *P ≤* 0.001), Pr-AKI (3-fold, *P* = 0.001) and maternal age were associated with the greatest increased risk for a reduced eGFR at first visit. HIV and Pr-AKI remained a significant risk of reduced eGFR at 1 year (Supplementary Table S3). The multivariate analysis demonstrated similar findings at first visit; however, only Pr-AKI was associated with a 5-fold (*P* = 0.013) increased risk for reduced eGFR at 1 year. HIV, despite an OR of 3.11 (95% CI: 0.51–19.04), lacked statistical significance at 1 year. Advanced maternal age was only found to be a significant predictor at first visit (*P* = 0.001) (Table 3).

Persistent albuminuria was a prominent finding in the cohort. On univariate analysis, at first visit, prior chronic hypertension, Pr-AKI, and prior HDP all doubled the risk of persistent albuminuria (*P* = 0.018, *P* = 0.032, and *P* = 0.017, respectively). At 1-year follow-up, Pr-AKI (OR: 3.35, *P* = 0.013) remained a significant risk factor on univariate analysis (Supplementary Table S3). Only Pr-AKI was a significant risk factor for albuminuria at first visit and 1-year on multivariate analysis (Table 3).

**Figure 3:**
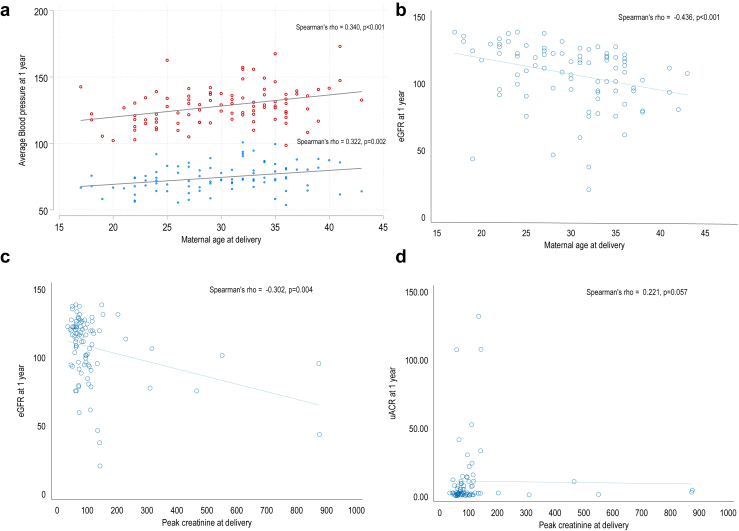
This figure depicts the correlation between variables associated with primary events of hypertension, reduced eGFR and albuminuria. (a) Correlating maternal age with systolic (red) and diastolic (blue) blood pressure. The graph shows a positive correlation between maternal age and systolic (red) and diastolic (blue) blood pressure. (b) Demonstrates that advancing maternal age was correlated with a reduced eGFR at 1 year. (c) Demonstrates that a higher creatinine at delivery correlated with a reduced eGFR at (c) and (d) persistent albuminuria, at 1 year. eGFR, estimated glomerular filtration rate; uACR, urinary albumin-to-creatinine ratio.

There was a positive correlation between highest systolic and diastolic BP at delivery and mean annual systolic and diastolic BP at follow-up. Furthermore, maternal age at delivery was positively correlated with BP (Figure 3a), and negatively with eGFR (Figure 3b). Higher creatinine at delivery correlated negatively with eGFR (Figure 3c) and albuminuria (Figure 3d) at 1 year.

## Discussion

This study adds to the limited available data on the long-term outcomes of women following preeclampsia in a resource-limited setting. We identified the following: (i) a high proportion of incident hypertension and albuminuria, with a large proportion of women with impaired kidney function that persisted at follow-up; (ii) raised BMI was a significant risk factor on multivariate analysis for incident hypertension; and (iii) Pr-AKI at delivery was a risk factor for reduced eGFR and persistent albuminuria at 1 year.

### Limited Data on CKD following HDP in Africa

There is a convincing body of literature that preeclampsia is associated with a future risk of albuminuria, CKD, and end-stage kidney disease.^14^, ^15^, ^16^^,^^26^ The pathophysiology of preeclampsia and the hypothesized mechanisms linked to future CKD have been well-described.^26^ The angiogenic imbalance caused by preeclampsia leads to endothelial dysfunction and podocyte injury, both predisposing to future CKD.^26^^,^^27^ Direct kidney injury may be further compounded by peri-partum hypovolemia as a result of postpartum hemorrhage or sepsis as well as the intravascular depletion that occurs in the context of severe preeclampsia. In addition, international data from high-income countries demonstrate a clear association between HDPs and increased risk of future cardiovascular disease and metabolic (atherogenic lipid profile and diabetes) derangement.^28^^,^^29^ The increased risk of hypertension and diabetes following preeclampsia, further compromises kidney health in the future.^8^^,^^11^^,^^26^^,^^29^

Despite the high burden in Africa, little is known about the prevalence of long-term complications in this low-income setting. To our knowledge, only 4 studies, with short duration of follow-up (42 days to 1 year), have reviewed sustained proteinuria and deranged kidney function following preeclampsia (Supplementary Table S4).^30^, ^31^, ^32^, ^33^ However, the studies had relatively short duration of follow-up, discrepant methods of proteinuria analysis (including dipstick analysis), and lacked exclusion criteria for CKD.

In our study, we excluded CKD and provide longer-term follow-up data on kidney function and albuminuria. Roughly a fifth of the cohort had a sustained reduction in eGFR (< 90 ml/min per 1.73 m^2^) at first visit and 1 year. Similar to international data, we found that advanced maternal age increased the risk for CKD at first visit.^32^^,^^34^ The well-described association of preterm delivery and small for gestational age and CKD was not found in our cohort.^35^^,^^36^ This is likely because of the severity of the preeclampsia and eclampsia, with a high proportion of prematurity and severe growth restriction in the cohort, overall. Raised BMI was not associated with CKD or albuminuria in our cohort, likely because of the short term follow-up of only 1 year. This contrasts international literature, which describes an association between BMI and CKD; however, these studies had longer follow-up periods.^35^ Little is known on how HIV impacts long-term recovery from preeclampsia. It has been shown that following preeclampsia, people with HIV have a higher odds (OR: 1.68, 95% CI 1.09–2.60, *P* = 0.019) of persistent hypertension at follow-up compared with HIV negative women, even after accounting for age, BMI, and duration since delivery.^37^ Our study showed a 4-fold (OR: 4.33, 95% CI: 1.73–10.84; *P* = 0.002) increased risk for kidney dysfunction at first follow-up visit in people with HIV on multivariate analysis, but no increased risk for hypertension. Owing to small numbers at 1-year follow-up, this lost statistical significance. However, this concerning risk needs further investigation with larger numbers and longer follow-up duration.

### Burden of Pr-AKI Associated With Preeclampsia in Africa

High rates of Pr-AKI due to severe preeclampsia were identified in our cohort. Pr-AKI is prevalent in low- and low-middle income countries and the commonest causes include preeclampsia, hemorrhage, and sepsis.^7^^,^^38^ Unacceptably high mortality rates for mother and fetus are reported in pregnancies complicated by Pr-AKI.^7^^,^^38^ The limited literature from Africa of Pr-AKI in preeclampsia are summarized in Supplementary Table S5. Despite Pr-AKI being a recognized risk factor for CKD,^39^^,^^40^ only 3 of the 21 studies reported on full kidney recovery, which ranged from 62% to 87%.^17^^,^^33^^,^^41^

In our study, which classified AKI using the Kidney Disease: Improving Global Outcomes classification, only 67% of women recovered completely by 1 year. We demonstrated a strong association between Pr-AKI and the risk of sustained reduction in eGFR, with a 3-fold risk at first visit (*P* = 0.002) and 5-fold risk at 1 year (*P* = 0.013). In addition, it was associated with sustained albuminuria at 1 year (*P* = 0.015). Rates of Pr-AKI in high-income countries are lower, reflecting heightened surveillance and timely management. However, there is a high prevalence of Pr-AKI in low- and lower-middle-income countries and serves as an ideal opportunity for task sharing with midwifery. Early recognition, prompt delivery, avoidance of further kidney insults, and careful fluid management may provide a cost-effective approach to mitigate long term CKD.

### Hypertension Following HDP in Africa

In Supplementary Table S6, we demonstrate prevalence of hypertension following preeclampsia in African studies. The majority of these involved prospective follow-up. Hypertension ranged from 17% to 62% in the first 6 months postpartum,^37^^,^^42^ and remained high after 1 year (24%–61%).^42^^,^^43^ Our study echoes these findings, reporting high rates of incident hypertension at first visit, 1 and 2 years at 44%, 47%, and 45%, respectively. Risk factors for hypertension, in the African studies, included maternal age, low birthweight, higher creatinine and proteinuria at delivery, proteinuria at 6 weeks postpartum, prematurity, high BMI, multiparous, family history of hypertension, HIV, and severe preeclampsia.^31^^,^^32^^,^^37^^,^^42^, ^43^, ^44^ Our study demonstrated that a raised BMI was predictive of incident hypertension, whereas maternal age trended toward significance on multivariate analysis.

Of the women in our cohort who had a new diagnosis of hypertension, there was good treatment uptake, 76% were taking treatment at first follow-up visit and 96% at 1 year. In South Africa, only 23% of people with hypertension are reportedly on treatment.^45^ The high treatment uptake may reflect the selection bias of the cohort who attended follow-up. Nevertheless, this does highlight the importance of education around hypertension and is a potential area to focus on task-sharing with midwifery and primary health care providers.

### Recommendations for Improved Postpartum Care in a Lower-Income Setting

Postpartum follow-up is essential in these women because their long-term health outcomes are compromised relative to those with normotensive pregnancies. Poor outcomes are not limited to women in whom abnormalities persist but are also observed in those with an apparent initial recovery. Therefore, longer follow-up and integrated health care is essential. Initial management should include adequate antenatal care and education. Research has demonstrated that a minimum of 4 antenatal care visits providing education improves attendance postpartum.^46^^,^^47^

Point-of-care testing (blood and urine analysis) and integrated health care for mother and child is absolutely essential, especially in settings with limited or no laboratory access. Integration of maternal health into infant vaccination schedules is a potential solution where task sharing could occur between midwives and primary health care practitioners. This visit should incorporate basic screening (BP, dipsticks, and creatinine testing) for mothers. Lastly, educating primary health care and empowering mothers (FIGO passport)^48^ on future health risk postpartum is essential to improve care. In addition, advocacy for policy change to include coverage for postpartum care is vital (Table 4).

**Table 4:** Recommendations for postpartum care

Intrapartum:
• Regular ANC in pregnancy (minimum of 4 visits improves outcomes and follow-up)
Postpartum:
Integrating health care:
• Maternal and baby health checks at scheduled infant vaccination appointments.
• Availability of testing à POC testing
Empowering women:
• FIGO passport
• Educating women on their future risk
Empowering health care workers and primary health carers
• On early identification and recognition of the future renal -cardio-metabolic risk of women with complicated obstetric histories.
• Advocacy for policy change on postpartum care

ANC, Antenatal care; FIGO, International Federation of Gynaecology and Obstetrics; POC, point-of-care.

### Limitations

The strength of this study is the addition of long-term data from African women following preeclampsia. Nevertheless, the limitations include the high loss-to-follow-up rate. For those who did follow-up, there is potential for biased results, because these are women who chose to access health care and therefore potentially more likely to be taking treatment. There was referral bias into our follow-up service. Our hospital is a referral center for complicated preeclampsia; therefore, owing to limited staffing, only women with severe preeclampsia were selected for follow-up. In addition, despite attempting to exclude prior CKD from our cohort, albuminuria is not a routine test performed in our primary health care service, so we are unable to exclude the presence of albuminuria in the cohort prior to developing preeclampsia. Owing to attrition, there were smaller numbers of follow-up data at 1- and 2-years, this may have impacted statistical validity. The low numbers of 1-year follow-up data in patients with HIV limited meaningful longer term statistical analysis. This is an area that needs research in the future.

## Conclusion

This study provides valuable insights into the long-term health outcomes following severe preeclampsia in a low-resource setting. High rates of incident hypertension, kidney dysfunction, and microalbuminuria underscore the need for continuous monitoring and early intervention postpartum. The findings highlight the role of Pr-AKI in predicting future kidney outcomes and the importance of long term data to guide future clinical practice and policy development, particularly in resource-limited environments.

## Disclosure

All the authors declared no competing interests.

## Supplementary Material

Supplementary File (PDF) **Figure S1.** Describes the degree of uACR (mg/mmol) and eGFR at first visit and 1-year, aligned with KDIGO CKD definition. **Table S1.** Description of incident hypertension, persistent albuminuria, and kidney dysfunction at first visit, 1 and 2 years, comparing those with and without HIV, birth centile < 10, HELLP, and prior HDP definition. **Table S2.** Description of hypertension in the cohort at first visit, 1 and 2 years. **Table S3.** Univariant analysis of primary outcomes: incident hypertension, reduced eGFR, and persistent albuminuria. **Table S4.** African studies reviewing the long-term proportions of those with sustained kidney dysfunction and proteinuria. **Table S5.** Studies related to PRAKI secondary to hypertensive disorders of pregnancy. **Table S6.** African studies reviewing the long-term proportions of sustained hypertension. STROBE Checklist.

## Footnotes

Supplementary File (PDF)
**Figure S1.** Describes the degree of uACR (mg/mmol) and eGFR at first visit and 1-year, aligned with KDIGO CKD definition. **Table S1.** Description of incident hypertension, persistent albuminuria, and kidney dysfunction at first visit, 1 and 2 years, comparing those with and without HIV, birth centile < 10, HELLP, and prior HDP definition. **Table S2.** Description of hypertension in the cohort at first visit, 1 and 2 years. **Table S3.** Univariant analysis of primary outcomes: incident hypertension, reduced eGFR, and persistent albuminuria. **Table S4.** African studies reviewing the long-term proportions of those with sustained kidney dysfunction and proteinuria. **Table S5.** Studies related to PRAKI secondary to hypertensive disorders of pregnancy. **Table S6.** African studies reviewing the long-term proportions of sustained hypertension. STROBE Checklist.
