# Supplementary material for: Impact of Preeclampsia on Long-Term Kidney Function in a Low-Resourced Setting
Source: Kidney Int Rep. 2025 Nov 20;11(2):103689. doi: 10.1016/j.ekir.2025.11.014 (PMC12769136; doi:10.1016/j.ekir.2025.11.014)
Supplement: Supplementary File (PDF) — Figure S1. Describes the degree of uACR (mg/mmol) and eGFR at first visit and 1-year, aligned with KDIGO CKD definition. Table S1. Description of incident hypertension, persistent albuminuria, and kidney dysfunction at first visit, 1 and 2 years, comparing those with and without HIV, birth centile < 10, HELLP, and prior HDP definition. Table S2. Description of hypertension in the cohort at first visit, 1 and 2 years. Table S3. Univariant analysis of primary outcomes: incident hypertension, reduced eGFR, and persistent albuminuria. Table S4. African studies reviewing the long-term proportions of those with sustained kidney dysfunction and proteinuria. Table S5. Studies related to PRAKI secondary to hypertensive disorders of pregnancy. Table S6. African studies reviewing the long-term proportions of sustained hypertension. STROBE Checklist. [file mmc1.pdf]

Supplementary Figure 1: Describes the degree of uACR (mg/mmol) and eGFR at first visit and 1-year, aligned with KDIGO CKD definition

		A1		A2		A3	
		Normal or mildly increased		Moderately increased		Severely increased	
		uACR 0 – 2.9		uACR 3- 29.9		uACR >30	
	eGFR (ml/min/1.73^2)	First visit	1-year	First visit	1-year	First visit	1-year
G1	90 -max	75	33	68	20	30	2
G2	60 - 89	22	7	18	4	5	0
G3a	45-59	0	1	4	1	2	0
G3b	30 - 44	0	0	1	1	0	1
G4	15-29	0	0	0	0	0	1

uACR, albumin:creatinine ratio; eGFR, estimated glomerular filtration rate

Figure legend Supplementary figure 1: risk stratification of cohort at first visit (n=225) and 1-year (n= 71) for those who had both creatinine and uACR available. Note CKD Stages were made on only one eGFR assessment not KDIGO recommended two readings 3 months apart.

Supplementary table 1: **Description of incident hypertension, persistent albuminuria and kidney dysfunction at first visit, 1- and 2-years, comparing those with and without HIV, birth centile <10,HELLP and prior HDP**

Description of incident hypertension, persistent albuminuria and kidney dysfunction at first visit, 1- and 2-years, comparing those with and without HIV				
**Follow up data**	**Total cohort**	**HIV negative**	**HIV positive**	**P - value**
**First visit**	239	209	30	
Incident HPT[#]	75/170 (44%)	63/145 (43%)	12/25 (48%)	0.670
eGFR <90[*]	52/234 (22%)	37/204 (18%)	15/30 (50%)	<0.001
Albuminuria[*]	131/230 (57%)	118/201(59%)	13/29 (45%)	0.160
**1st Annual visit 1**	93	85	8	
Incident HPT[#]	28/59 (47%)	25/52 (48%)	3/7 (43%)	1.00
eGFR <90[*]	18/88 (20%)	14/80 (18%)	4/8 (50%)	0.052
Albuminuria[*]	34/74 (46%)	32/69 (46%)	2/5 (40%)	1.00
**2nd Annual visit 2**	34	32	2	
Incident HPT[#]	9/20 (45%)	9/18 (50%)	0/2 (0%)	-
eGFR <90[*]	4/31 (13%)	3/29 (10%)	1/2 (50%)	-
Albuminuria[*]	18/32 (56%)	17/31 (55%)	1/1(100%)	-
**Description of incident hypertension, persistent albuminuria and kidney dysfunction at first visit, 1- and 2-years, comparing those born with and without a birth centile <10**				
**Follow up data**	**Total cohort**	**Pre-eclampsia with birth centile >10**	**Pre-eclampsia with birth centile <10**	**P - value**
**First visit**	229	95	134	
Incident HPT[#]	73/164 (45%)	29/69 (42%)	44/95 (46%)	0.590
eGFR <90[*]	50/225 (22%)	18/93 (19%)	32/132 (24%)	0.390
Albuminuria[*]	125/222 (56%)	48/92(52%)	77/130 (59%)	0.300
**1st Annual visit 1**	90	37	53	
Incident HPT[#]	27/57 (47%)	12/23 (52%)	15/34 (44%)	0.550
eGFR <90[*]	17/84 (20%)	7/31 (23%)	10/53 (19%)	0.680
Albuminuria[*]	30/70 (43%)	9/26 (35%)	21/44 (48%)	0.330
**2nd Annual visit 2**	33	14	19	
Incident HPT[#]	9/20 (45%)	5/9 (56%)	4/11 (36%)	0.390
eGFR <90[*]	4/30 (13%)	2/13 (15%)	2/17 (12%)	1.000
Albuminuria[*]	17/31 (55%)	4/12 (33%)	13/19 (68%)	0.075
**Description of incident hypertension, persistent albuminuria and kidney dysfunction at first visit, 1- and 2-years, comparing those born with and without HELLP**				
**Follow up data**	**Total cohort**	**PE without HELLP**	**PE with HELLP**	**P - value**
**First visit**	240	144	96	
Incident HPT[#]	76/170 (45%)	44/93 (47%)	32/77 (42%)	0.450
eGFR <90[*]	52/235 (22%)	27/140 (19%)	25/95 (26%)	0.200
Albuminuria[*]	131/231(57%)	78/139 (56%)	53/92 (58%)	0.820
**1st Annual visit 1**	94	59	35	
Incident HPT[#]	28/59 (47%)	20/34 (59%)	8/25 (32%)	0.041
eGFR <90[*]	18/88 (20%)	9/54 (17%)	9/34 (26%)	0.290
Albuminuria[*]	34/74 (46%)	22/46 (48%)	12/28 (43%)	0.680
**2nd Annual visit 2**	34	23	11	
Incident HPT[#]	9/20 (45%)	6/12 (50%)	3/8 (38%)	0.580

eGFR <90*	4/31 (13%)	2/22 (9%)	2/9 (22%)	0.560
Albuminuria*	18/32 (56%)	12/22 (55%)	6/10 (60%)	1.00
**Description of sustained hypertension, persistent microalbuminuria and kidney dysfunction at first visit, 1- and 2-years, comparing those with and without prior HDP.**				
**Follow up data**	**Total cohort**	**No prior HDP**	**Prior HDP**	**P - value**
**First visit**	**234**	**176**	**58**	
Incident HPT#	73/165 (44%)	62/148 (42%)	11/17 (65%)	0.073
eGFR <90*	50/229 (22%)	32/171 (19%)	18/58 (31%)	0.050
Microalbuminuria*	127/225 (56%)	87/168 (52%)	40/57 (70%)	0.016
**1st Annual visit 1**	89	64	25	
incident HPT#	24/55 (44%)	22/51 (43%)	2/4 (50%)	0.790
eGFR <90*	16/83 (19%)	9/58 (16%)	7/25 (28%)	0.190
Microalbuminuria*	30/69 (43%)	20/47 (43%)	10/22 (46%)	0.820
**2nd Annual visit 2**	32	22	10	
incident HPT#	7/18 (39%)	7/17 (41%)	0/1 (0%)	1.00
eGFR <90*	4/29 (14%)	1/19 (5%)	3/10 (30%)	0.100
Microalbuminuria*	17/30 (57%)	10/20 (50%)	7/10 (70%)	0.440

HIV; human immunodeficiency virus, HELLP; haemolysis elevated liver enzymes and low platelets, HPT; hypertension, eGFR; estimated glomerular filtration rate (ml/min/1.73m^2),

*Denominator reflects available data

#Incident hypertension reflects new onset of HPT following pre-eclampsia. Analysis therefore removed those known with chronic hypertension

Supplementary Table 2: Description of hypertension in the cohort at first visit , 1- and 2-years.

	Cohort (n=241)	PE without AKI (n=122)	PE with AKI (n=119)	P value
**First visit**	**n=241**	**n=122**	**n=119**	
Systolic (mmHg)	134 +/- 20	136 +/-20	132 +/- 20	0.180
Diastolic (mmHg)	79 +/- 14	80 +/-15	79 +/- 14	0.360
Pulse	84 +/- 13	86 +/-13	82 +/- 14	0.016
Chronic HPT	68/241 (28%)	35/122 (29%)	33/119 (28%)	0.864
Incident HPT[#]	76/171 (44%)	40/86 (47%)	36/85 (42%)	0.580
HPT at V1[*]	128/241 (53%)	67/122 (55%)	61/119 (51%)	0.570
On treatment	97/128 (76%)	52/67 (76%)	45/61(74%)	0.794
**Visit year 1**	**n=93**	**n=51**	**n=42**	
Systolic (mmHg)	127 +/- 14	129 +/- 13	125 +/- 15	0.220
Diastolic (mmHg)	75 +/- 11	76 +/-10	74 +/- 12	0.340
Pulse	81 +/- 14	84 +/-14	77 +/- 12	0.017
Incident HPT[#]	28/60 (47%)	19/32(59%)	9/28 (33%)	0.046
HPT at Y1[*]	55/93 (59%)	34/ 51 (67%)	21/42 (50%)	0.100
On treatment	51/55 (93%)	32/34 (94%)	19/21 (90%)	0.585
**Visit year 2**	**n=34**	**n=14**	**n=20**	
Systolic (mmHg)	126 +/- 14	126 +/-16	125 +/- 13	0.870
Diastolic (mmHg)	73 +/- 11	72 +/-10	74 +/- 12	0.700
Pulse	82 +/- 14	84 +/-12	80 +/- 15	0.520
Incident HPT[#]	9/20 (45%)	5/9 (56%)	4/11 (36%)	0.390
HPT at Y2[*]	20/34 (59%)	9/14 (64%)	11/20 (55%)	0.590
On treatment	20/20 (100%)	9/9 (100%	11/11 (100%)	.

PE; pre-eclampsia, AKI; acute kidney injury, HPT; hypertension, mmHg; millimetres mercury, V1; first visit, Y1; year 1 visit, Y2; year 2 visit.

[*]Patients on treatment and/ or BP >140/90mmHg

[#]Incident hypertension reflects new onset of HPT following pre-eclampsia. Analysis therefore removed those known with chronic hypertension

Supplementary Table 3: Univariant analysis of primary outcomes: incident hypertension, reduced eGFR and persistent albuminuria

	Incident HPT 3 first visit			Incident HPT at 1 year		
	**OR**	**CI**	**p-value**	**OR**	**CI**	**p-value**
Age at delivery	1.07	1.02-1.13	0.005	1.18	1.06 – 1.32	0.002
BMI >30	1.55	0.83 – 2.92	0.172	4.40	1.44 – 13.80	0.009
HIV	1.20	0.51 – 2.81	0.672	0.81	0.16 – 3.98	0.795
Prior HDP	2.54	0.89 – 7.24	0.081	1.32	0.17 – 10.11	0.790
<34 weeks	0.94	0.50 -1.77	0.840	0.94	0.34 – 2.61	0.902
SGA	1.19	0.64 – 2.22	0.586	0.72	0.25 – 2.09	0.551
GA	1.01	0.95 – 1.08	0.742	0.95	0.83 – 1.09	0.496
HELLP	0.79	0.43 – 1.46	0.453	0.33	0.11 – 0.97	0.044
Abruptio	1.20	0.51 – 2.81	0.672	-	-	-
Pr-AKI at delivery	0.84	0.46 – 1.55	0.584	0.34	0.12 – 0.99	0.049
	**Reduced eGFR < 90 at first visit**			**Reduced eGFR < 90 at 1-year**		
	**OR**	**CI**	**p-value**	**OR**	**CI**	**p-value**
Age at delivery	1.12	1.06 – 1.18	<0.001	1.08	0.99 – 1.19	0.087
BMI >30	1.42	0.75 – 2.68	0.287	1.06	0.37 – 3.08	0.914
HIV	4.51	2.03 – 10.04	<0.001	4.71	1.05 – 21.15	0.043
Prior HDP	1.95	0.99-3.84	0.052	2.11	0.69 – 6.53	0.192
<34 weeks	1.61	0.81 -3.18	0.171	1.59	0.54 – 4.72	0.403
SGA	1.33	0.70 – 2.56	0.386	0.80	0.27 – 2.37	0.683
GA	0.97	0.91 – 1.04	0.389	0.94	0.83-1.06	0.295
HELLP	1.49	0.81 – 2.78	0.204	1.80	0.63 – 5.12	0.207
Abruptio	0.98	0.37 – 2.55	0.960	2.29	0.51 – 10.26	0.281
Chronic HPT	0.25	0.90 - 3.30	0.098	1.60	0.56 - 4.60	0.383
Pr-AKI at Delivery	3.04	1.58 – 5.86	0.001	4.40	1.41 – 13.75	0.011
	**Albuminuria at first visit**			**Albuminuria at 1-year**		
	**OR**	**CI**	**p-value**	**OR**	**CI**	**p-value**
Age at delivery	1.02	0.98 – 1.06	0.272	1.06	0.97- 1.15	0.183
BMI >30	1.54	0.89 -2.63	0.117	2.22	0.85 – 5.83	0.104
HIV	0.57	0.26 - 1.25	0.162	0.77	0.12 - 4.91	0.783
Prior HDP	2.19	1.15 – 4.17	0.017	1.13	0.41 – 3.12	0.821
<34 weeks	1.04	0.60 – 1.79	0.889	1.83	0.72 – 4.68	0.205
SGA	1.33	0.78 – 2.28	0.297	1.72	0.63 – 4.69	0.286
GA	0.97	0.92 – 1.03	0.362	0.91	0.82 – 1.02	0.097
HELLP	1.06	0.62 – 1.81	0.823	0.82	0.32 – 2.11	0.678
Abruptio	0.63	0.28 – 1.39	0.253	0.45	0.82 – 2.50	0.362
Chronic HPT	2.11	1.13 -3.91	0.018	1.41	0.55 - 3.61	0.475
Pr-AKI at Delivery	1.78	1.05 – 3.01	0.032	3.35	1.29 – 8.74	0.013

BMI; body mass index, HIV; human immunodeficiency virus, HDP; hypertensive disorders in pregnancy, SGA; small for gestational age, GA; gestational age, HELLP; haemolysis elevated liver enzymes and low platelets, HPT; hypertension, Pr-AKI; pregnancy related acute kidney injury

Supplementary Table 4: African studies reviewing the long term proportions of those with sustained kidney dysfunction and proteinuria.

	Ndayambagye EB et al, (2010)	Kaze, FF et al, (2014)	Muteke, K et al, (2023)	Ishuka, SM (2021)[1]	Our study
	**Uganda (2010)[2]**	**Cameroon (2014)[3]**	**Uganda (2023)[4]**	**Nigeria (2021)**	**South Africa (2025)**
**Study design**	N= 195<br>(2008 – 2009)<br>Prospective FU	N= 54<br>(2010 – 2012)<br>Prospective FU	N=86<br>(2017 – 2018)<br>Prospective FU	N= 410 HDP; n=78 normotensive<br>(2027 – 2018)<br>Prospective FU	N=241<br>(2020 – 2024)<br>Prospective FU
**Breakdown**	25 (12.8%) mild PE<br>160 (82.1%) severe PE<br>10(5.1%) EC	37 (68.5%)<br>17 (31.5%)	86 (100%) PE	N=33 (9%) chronic HPT<br>N=75 (21%)<br>N= 198 (55%) PET<br>N=56 (15%) EC	Severe PET (95%)<br>EC (5%)
**Follow - up**	6 weeks	6 weeks, 3/12 and 6 months	Day 1, 7, 21 and 42	9 weeks, 6 months, 1 year	3 -, 12- and 24 months
**LTFU**	2 died<br>3 LTFU	3 died<br>2 LTFU	Not stated	147(36%) at 9/52<br>178(43%) at 6/12<br>132(32%) at 1-year	90/183 (49%) at 1-year<br>37/71 (52%) at 2-years
**Outcomes**					
**Persistent HPT**	141 (72,3%)	6/52 : 23 (42.6%)<br>3/12: 15 (27.8%)<br>6/12: 8 (14.8%)	5 (6.9%) at 42 days		3 months – 44%<br>12 months – 47%<br>24 months – 45%
**Persistent proteinuria**	44.4% of persistent HPT had degree of proteinuria. (3+dipstix)<br><br>Persistent HPT: (9.3%) Vs Normotensive (2.9%)	6/52 : 26 (48.1%)<br>3/12: 17 (31.5%)<br>6/12: 1 (18%)	13 (15.1%) at 42 days on dipsticks	Not reported	Microalbuminuria<br>3 months – 55%<br>12 months – 42%<br>24 months – 40%
**Renal dysfunction**	Higher mean Cr at delivery in the persistent HPT vs. normotensive.	Renal function normalised in all women within 3/12.	54/86 (62.8%) at day 1<br>33/86 (38.4%) at 42 days	eGFR <60<br>6 months: 4.3%<br>1 year: (3.5% overall)	eGFR < 90ml/min/m<br>3 months – 22%<br>12 months – 20%<br>24 months – 13%
**Risk factor for outcomes**	**Risk factors associated with persistence HPT:**<br>• age >30-34 yr (p= 0,001)<br>• Serum creatinine at admission (p = 0,001)<br>• urine protein at 6/52 (p=0,051)	**Risk factors for persistent HPT at 3/12:**<br>• Advanced age,<br>• higher BMI,<br>• low GA,<br>• LBW,<br>• proteinuria at delivery<br><br>**Composite outcome hypertension or proteinuria**<br>• Advanced age<br>• higher BMI	Time to resolution of HPT was 3.5x faster in primigravida than in multiparous women.	Gestational age at 6/12	**On multivariate analysis risk factors at one year for HPT:**<br>- advanced maternal age<br><br>**Risk factors – at one year for reduced eGFR and microalbuminuria:**<br>- AKI

N, number; FU, follow-up; HDP, hypertension disorders of pregnancy; PE, pre-eclampsia; EC, eclampsia; HPT, hypertension; GH, gestational hypertension; Cr, creatinine; BP, blood pressure; BMI, body mass index; OR, odds ratio; CI, confidence interval; ARR, absolute relative risk; CRR, crude relative risk; RR, relative risk; Fam Hx, family history; Sys, systolic; dias, diastolic; EOPET, early onset of pre-eclampsia; GA, gestational age; HELLP, haemolysis, elevated liver enzymes and low platelets;

Supplementary Table 5 : studies related to PRAKI secondary to hypertensive disorders of pregnancy

	Study design	PRAKI n (%)	No PET/ total cohort	AKI stage 3	Dialysis	Mortality in PRAKI	Full kidney recovery	Perinatal mortality
**Pre- eclampsia**								
Conti-Ramsden et al, South Africa (2019)	Prospective cohort study (PET)	237[K] (15%)	1547	4.1%[K]	12.5%	3% in total cohort	80,4%	SB (RR 2.2). Relative risk increased with AKI and AKI severity
Nathan et al, South Africa (2018)	Prospective cohort study (PET)	272[K] (18%)	1547	-	-	3% with AKI (compared to 0.5% with no AKI)	-	SB (85%)
Muteke et al, Uganda (2023)	Prospective cohort study (PET)	97	-	-	-	--	@ 42 days 38.4% not recovered	-
Ngeufack et al, Cameroon (2017)	Prospective cross-sectional study (PET)	12/170[x] (7%)	2148	-	-	-	-	SB (14%)<br>Prem (10%), BW <2500g (88%)
Adu Bonsaffoh et al, Ghana (2014)	Prospective cross-sectional study (HDP)	3/ 368[X] (0.8%)	368 HDP/1856	-	-	3% in total cohort	-	-
Ugwa et al, Nigeria (2022)	Retrospective study (PET)	2%	426 PET /4181	-	-	4% in total cohort	-	Prem (23%), BW<2500g (9.6%)<br>SB (9%), END (8%)
Beyuo et al GHANA (2021)	PET Randomized control trial 12 HR VS 24 HR Magnesium Sulphate treatment	125/ 1176[X] (11%)	1176 PET /9500	-	-	0.4% in total cohort	-	Prem <32 16.7%, BW<1500g (19%)<br>SB (12%), END (20%)
Seyom et al, Ethiopia (2015)	Retrospective study (PET)	8[X] (7%)	121 HDP /5415	-	-	0	-	SB (10%), END (2%)<br>Prem (31%)
**Severe Pre-eclampsia or eclampsia**								
Berhe et al, (2024)	Retrospective study of PRAKI	187/27350 (0.7%)	75.4% HDP	-	16 (8.6%)	8% in total cohort	162 (87%)	SB 33 (18%)<br>IUFD 20 (11%)
Hassan et al, Uganda (2022)	Prospective cohort study severe (PET/EC)	30/70[K] (43%)	4547	0%	-	-	-	-
Jido et al Nigeria (2012)	Prospective cohort study (EC)	6/120[X] (5%)	120/ 10263 Incidence EC 1.2%	-	1/120	12% in total cohort	-	SB (23%)<br>BW<2500g (26%)

Irene et al, Kenya (2021)	Prospective cohort study (EC)	8[X] (15%)	53			0	6-week F/U no data on resolution AKI	END 5<br>BW <2500g (21%)
Adamu et al, Nigeria (2012)	Retrospective study (EC)	117[X] (11%)	1027 EC/ 23266 Incidence 4.4%	-	-	18% in the total cohort	-	SB (81%)<br>END (18.9%)
Ngwenya et al, Zimbabwe (2017)	Retrospective analysis (PET/EC)	2/121[K] (2%)	121/9086 Incidence 1.3%	-	-	2% in total cohort	-	SB + END (50%)
Nyirenda et al, Zambia (2019)	Prospective cohort study (Severe PET)	5%[X]	175/?	-	-	2% in total cohort	-	SB (28%)
Priso et al, Cameroon (2015)	Retrospective review (PET/ET in ICU)	30/74 [X] (67%)	74			24% in total cohort		-
Tadese, M et al, Ethiopia (2023)	Cross sectional study Severe (PET)	4% [X]	384	-	-	0.9% in total cohort	-	-
Drakeley et al, (2001)	Retrospective study	72	5200	47% (stage 2 + 3)	7 (10%)	0	-	
Igberase et al, Nigeria (2006)	Retrospective study	?	123/5242 2.3% EC	-	-	47% due to renal failure		Perinatal mortality rate 195/1000
Onuh et al, Nigeria (2004)	Retrospective study (EC)	3/103[X] (3%)	103/ 7835 Incidence 1,32%	-	-	11%	-	Perinatal mortality rate 214/100000<br>Prem (68%)
Mostafa et al, Egypt (2021)	Prospective cohort study (severe PET)	10/204[X] (5%)	204	-	-	0%		Prem (69%)<br>SB (13%)

HDP; hypertensive disorders of pregnancy, PET; pre-eclampsia, EC; eclampsia, ICU; intensive care unit; AKI; acute kidney injury, [x] unknown method of determining AKI, [K] KDIGO criteria used, F/U; follow-up, SB; stillbirth, END; early neonatal death, Prem; premature, IUFD; intrauterine fetal death, BW; birth weight

Supplementary Table 6: African studies reviewing the long term proportions of sustained hypertension

	Nakimuli, A et al, (2013)	Nganou-Gnindigo, C et al , (2021)	Mooij, R et al, (2021)	Ishaku, S et al, (2021)	Fadalallah ZM, et al, (2016)	Amougou SN et al, (2019)	Ahmed, A et al (2023)	Lugobe, H et al (2023)	Mukosha, M et al (2024)
	Uganda	Cameroon	Tanzania	Nigeria	Sudan	Cameroon	Tanzania	Uganda	Zambia
**Study design**	N=188<br>(2009 – 2011)<br>Prospective cohort	N= 92,<br>(2011 – 2016)<br>Prospective FU	N=24<br>Mixed methods questionnaire + interview	N= 287,<br>(2017 – 2019),<br>Prospective FU	N=165,<br>(March – Oct 2014)<br>Prospective cohort	N=136<br>(2009 – 2016)<br>Retrospective review	N = 309<br>Multicentre study<br>5 died postpartum, 12 LTFU	(n=111)<br>Prospective cohort<br>(Jan 2019 – Dec 2019)	N=136<br>44 HIV, 92 HIV negative<br>(Jan2022 – June 2023)
**Break down**			9 – PE<br>15 - EC		136 (82.4%) mild PE<br>29 (17.6%) severe PE	136 (100%) PE	292	5-GH<br>81- PE<br>25-EC	136 - PE
**Follow - up**	3 months	6 months	6-7 years post delivery	6/12 and 1 year	6 weeks	1 year	3 months	3 months	6/52, 3-months and 6-months
**Outcomes**									
**Persistent HPT**	64 (34%)	30 (32.6%)	17% overall<br>29% in those after severe PE<br><br>56% described anxiety	In gestational HPT:<br>6/12: (22%)<br>1yr (22 %)<br><br>In PE<br>6/12: 62%<br>1 yr: 61%	58 (35.2%)	32 (23.5%)<br><br>Incidence rate 2.85%/ year	120 (41.1%)	21/53 (39%)	6/52 37.4%<br>3/12 17.1%<br>6/12 16.9%
**Risk factor for outcomes**	**Risk factor for persistent HPT:**<br>• Age > 25 (CRR 1.5). once age >30 crude risk ratio 4.43 (1.30 – 15. 09) 0.017.	1 in 3 women with PET/ EC develop persistent HPT<br><br>Risk factors for persistent HPT:	Women who suffered from (severe pre-) eclampsia may experience long-term sequelae, including hypertension,	**Univariate risk factors for persistent HPT:**<br>• maternal age, [ORs =1.11/year (95% CI; 1.06–1.15	Severe PE were 7.3-times more likely to experience persistent hypertension than patients with mild pre-eclampsia (95%	**Risk factors associated with persistent HPT:**<br>• age ≥ 40 years (OR = 20.7 (1.1 - 390.0); p = 0.043),	**Strongest indicator for persistent HPT:**<br>• renal dysfunction at delivery		Higher odds (adjusted odds ratio [aOR] = 1.68, 95% CI: 1.09–2.60) of persistent hypertension among the HIV +treatment

	• Multiparous (CRR=)1<br>• GA 24 – 34/40 (CRR=1)<br>• HIV status (CRR 10.95, p=0.03)<br>• Severe PE (CRR 3.8, p=0.001)	• Maternal age > 30 yes (OR 6.03, p=0.03)<br>• > 5 deliveries (OR 1.5, p=0.008)<br>• Fam Hx DM (OR 14.8, p=0.003)	depression and anxiety	• BMI, [1.03/kg/m^2 (95% CI; 1.00–1.07],<br>• sys and dias HPT at delivery), 1.01/mmHg (95% CI; 1.00–1.02) and 1.02/mmHg (95% CI; 1.00–1.03)<br><br>**Multivariable risk factors for persistent HPT**<br>• maternal age [OR 1.07/year (95% CI; 1.02–1.13]<br>• BMI at delivery 1.06/kg/m^2 (95% CI; 1.01–1.1)]	CI 1.6–32.2; P = 0.008)	• housewife profession (OR = 21,.8 (3,4-138.3); p = 0.001),<br>• gravidity > 4 (OR = 7.9 (1.0 - 59.1); p = 0.044),<br>• EOPE < 34 weeks' gestation (OR = 9.3 (2.1 ' 42.0); p = 0.004),<br>• Family Hx HPT (OR = 6.7 (1.0 - 44.2); p = 0.047)<br>• obesity (OR = 16.5 (2.3 - 120.6); p = 0.006).			group than HIV-negative counterparts after accounting for age, BMI and time since delivery.

N, number; FU, follow-up; HDP, hypertension disorders of pregnancy; PE, pre-eclampsia; EC, eclampsia; HPT, hypertension; GH, gestational hypertension; Cr, creatinine; BP, blood pressure; BMI, body mass index; OR, odds ratio; CI, confidence interval; ARR, absolute relative risk; CRR, crude relative risk; RR, relative risk; Fam Hx, family history; Sys, systolic; dias, diastolic; EOPET, early onset of pre-eclampsia; GA, gestational age; HELLP, haemolysis, elevated liver enzymes and low platelets;

STROBE Statement—Checklist of items that should be included in reports of ***cohort studies***

	Item No	Recommendation	Page No
**Title and abstract**	1	(*a*) Indicate the study's design with a commonly used term in the title or the abstract<br>(*b*) Provide in the abstract an informative and balanced summary of what was done and what was found	Pg 2<br>Pg 2
**Introduction**			
Background/rationale	2	Explain the scientific background and rationale for the investigation being reported	Pg 3-4
Objectives	3	State specific objectives, including any prespecified hypotheses	Pg 4
**Methods**			
Study design	4	Present key elements of study design early in the paper	Pg 4
Setting	5	Describe the setting, locations, and relevant dates, including periods of recruitment, exposure, follow-up, and data collection	Pg 4
Participants	6	(*a*) Give the eligibility criteria, and the sources and methods of selection of participants. Describe methods of follow-up<br>(*b*) For matched studies, give matching criteria and number of exposed and unexposed	Pg 5
Variables	7	Clearly define all outcomes, exposures, predictors, potential confounders, and effect modifiers. Give diagnostic criteria, if applicable	Pg 6/7
Data sources/ measurement	8*	For each variable of interest, give sources of data and details of methods of assessment (measurement). Describe comparability of assessment methods if there is more than one group	Pg 5
Bias	9	Describe any efforts to address potential sources of bias	
Study size	10	Explain how the study size was arrived at	Pg5
Quantitative variables	11	Explain how quantitative variables were handled in the analyses. If applicable, describe which groupings were chosen and why	Pg 7
Statistical methods	12	(*a*) Describe all statistical methods, including those used to control for confounding<br>(*b*) Describe any methods used to examine subgroups and interactions<br>(*c*) Explain how missing data were addressed<br>(*d*) If applicable, explain how loss to follow-up was addressed<br>(*e*) Describe any sensitivity analyses	Pg 7/8
**Results**			
Participants	13*	(a) Report numbers of individuals at each stage of study—eg numbers potentially eligible, examined for eligibility, confirmed eligible, included in the study, completing follow-up, and analysed<br>(b) Give reasons for non-participation at each stage<br>(c) Consider use of a flow diagram	Pg 8 Fig 1
Descriptive data	14*	(a) Give characteristics of study participants (eg demographic, clinical, social) and information on exposures and potential confounders<br>(b) Indicate number of participants with missing data for each variable of interest<br>(c) Summarise follow-up time (eg, average and total amount)	Pg 8 Table 1
Outcome data	15*	Report numbers of outcome events or summary measures over time	Pg8/9

Main results	16	(*a*) Give unadjusted estimates and, if applicable, confounder-adjusted estimates and their precision (eg, 95% confidence interval). Make clear which confounders were adjusted for and why they were included<br>(*b*) Report category boundaries when continuous variables were categorized<br>(*c*) If relevant, consider translating estimates of relative risk into absolute risk for a meaningful time period	Pg 10,11
Other analyses	17	Report other analyses done—eg analyses of subgroups and interactions, and sensitivity analyses	Pg 10,11,12
**Discussion**			
Key results	18	Summarise key results with reference to study objectives	Pg12
Limitations	19	Discuss limitations of the study, taking into account sources of potential bias or imprecision. Discuss both direction and magnitude of any potential bias	Pg 16
Interpretation	20	Give a cautious overall interpretation of results considering objectives, limitations, multiplicity of analyses, results from similar studies, and other relevant evidence	Pg 13-15
Generalisability	21	Discuss the generalisability (external validity) of the study results	Pg 16
**Other information**			
Funding	22	Give the source of funding and the role of the funders for the present study and, if applicable, for the original study on which the present article is based	Pg 16

*Give information separately for exposed and unexposed groups.

Note: An Explanation and Elaboration article discusses each checklist item and gives methodological background and published examples of transparent reporting. The STROBE checklist is best used in conjunction with this article (freely available on the Web sites of PLoS Medicine at http://www.plosmedicine.org/, Annals of Internal Medicine at http://www.annals.org/, and Epidemiology at http://www.epidem.com/). Information on the STROBE Initiative is available at http://www.strobe-statement.org.
